# Real-World Efficacy and Tolerability of Brigatinib in Patients with Non-Small Cell Lung Cancer with Prior ALK-TKIs in the United States

**DOI:** 10.1093/oncolo/oyac116

**Published:** 2022-07-04

**Authors:** Mohammad Jahanzeb, Huamao M Lin, Yanyu Wu, Pingkuan Zhang, Magdaliz Gorritz, Catherine B McGuiness, Wei-Ti Huang, Kainan Sun, Chi-Chang Chen, D Ross Camidge

**Affiliations:** Florida Atlantic University, Boca Raton, FL, USA; Takeda Development Center Americas, Inc., Lexington, MA, USA; Takeda Development Center Americas, Inc., Lexington, MA, USA; Takeda Development Center Americas, Inc., Lexington, MA, USA; IQVIA, Inc., Wayne, PA, USA; IQVIA, Inc., Wayne, PA, USA; IQVIA, Inc., Wayne, PA, USA; IQVIA, Inc., Wayne, PA, USA; IQVIA, Inc., Wayne, PA, USA; University of Colorado, Aurora, CO, USA

**Keywords:** ALK+ NSCLC, ALK-TKI, brigatinib, time-to-treatment discontinuation, adherence, real-world evidence

## Abstract

**Background:**

Real-world evidence for brigatinib, a next-generation anaplastic lymphoma kinase-tyrosine kinase inhibitor (ALK-TKI) used in ALK-rearranged non-small cell lung cancer, is scarce. This retrospective study evaluated real-world brigatinib utilization in the US post other ALK-TKIs.

**Materials and Methods:**

Adults with ≥1 brigatinib claim (index date) between 1 April 2017 and 30 September 2020 in the IQVIA longitudinal pharmacy claims database were followed until dose reduction, discontinuation, or end of follow-up. Patients had ≥12 months pre– and ≥1-month post–index observations.

**Results:**

A total of 413 patients treated with brigatinib were analyzed. Over 80% received ≥1 prior ALK-TKI; alectinib and crizotinib were the most common (58.8% and 51.3% patients, respectively). The median follow-up was 8.4 months. The median time to treatment discontinuation (TTD) for brigatinib was 10.3 months (95% CI, 8.2-15.0), with 45% remaining on therapy at 12 months. The TTD was shortest (~8 months) in patients receiving both crizotinib and alectinib and longest in patients who received alectinib only prior to brigatinib (11.8 months). Adherence was high, with 92.7% of patients having a medication possession ratio of >80%. The mean dose compliance score was 1.0. Most patients reached the brigatinib dose of 180 mg/day (77%); 13.2% of patients had a dose reduction, with 89.3% and 84.6% continuing 180 mg/day therapy at 3 and 6 months, respectively.

**Conclusions:**

Brigatinib appears to be effective and well-tolerated in the real-world ALK+ NSCLC population in the US, showing benefit in patients after a next-generation ALK-TKI. Notably, dose reduction rates appeared markedly less than those seen in trials when most trial-related dose reductions were for asymptomatic laboratory abnormalities.

## Teaser

Considering the evolving treatment landscape for ALK+ non-small cell lung cancer (NSCLC), more data are needed for brigatinib in both the post-crizotinib and post-next generation settings, which could help clinicians make informed decisions and maximize patient benefits. This article analyzes the real-world treatment and dosing patterns of brigatinib in the US.

**Implications for Practice**

The results of the present study suggest that brigatinib has real-world durable clinical benefits and is well tolerated. Patients using brigatinib after another ALK-TKI stayed on therapy for a significant duration of time with high adherence to therapy observed. Dose reductions of brigatinib appeared to occur at a lower rate. Providers should be made aware of the evidence for brigatinib dosing, tolerability, and effectiveness in real-world clinical practice.

## Introduction

Lung cancer is the leading cause of cancer-related mortalities worldwide, contributing to ~1.8 million deaths in 2020.^1-3^ In the US, it is the third most common malignancy, imposing a significant burden in terms of lives lost and healthcare costs.^4,5^ In 2020 alone, an estimated 253,537 new lung cancer cases and 159,641 related deaths have been reported in the US.^3,6^ Non-small cell lung cancer (NSCLC) accounts for 85% of all lung cancers.^6^ Anaplastic lymphoma kinase (ALK) gene arrangement has been recognized as a driver mutation underlying the development of NSCLC, identified in 3-5% of cases and more common in younger and light/never-smoker patients with adenocarcinoma histotype.^7,8^ Over the last decade, the therapeutic landscape for ALK-positive tumors (ALK+ NSCLC) has witnessed a paradigm shift, with the advent of ALK-tyrosine kinase inhibitors (ALK-TKIs),^9^ substantially improved the disease prognosis and expanded the available therapeutic options. To date, the US Food and Drug Administration (FDA) has approved 5 ALK-TKIs targeting ALK+ NSCLC^9-12^: the first generation, crizotinib, and the next generation, including ceritinib, alectinib, brigatinib, and lorlatinib.^9^ This array of agents allows drug sequencing, to enhance treatment efficacy and extend patient benefits.

Brigatinib was initially approved in 2017 for patients resistant or intolerant to crizotinib. In 2020, it received full FDA approval for adult patients with metastatic ALK+ NSCLC as detected by an FDA approved test.^10^ The recommended dose for brigatinib is 90 mg orally once daily (first 7 days), followed by 180 mg orally once daily.^13^ In the registrational ALTA trial post-crizotinib, which compared 90 mg with the 90 then 180 mg step-up dosing regimen, the dose reduction rate from the final target dose for each arm in the trial was 7% and 29%, respectively.^14^ Similarly, in the first-line ALTA-1L trial which compared the step-up dosing regimen with crizotinib, the proportion of patients in the brigatinib arm who reached 180 mg was 94%, and the dose reduction rate for brigatinib after reaching 180 mg was 44%.^15^ While these numbers appear higher than with other agents in the same class, the question of whether dose reductions for asymptomatic laboratory abnormalities mandated in the trial would carry through to the real world was uncertain. In terms of efficacy, brigatinib has been shown to exhibit clinical efficacy in patients with ALK+ NSCLC refractory to crizotinib (objective response rate [ORR], 45-56% and PFS, 16.7 months). In a recent Japanese study, brigatinib demonstrated significant efficacy and a well-tolerated safety profile in patients with ALK+ NSCLC post-alectinib (ORR, 34% and PFS, 7.3 months).^16^ In addition, in ALTA-1L brigatinib demonstrated strong clinical benefit as a first-line treatment versus crizotinib (PFS by BIRC: hazard ratio, 0.49 [95% CI, 0.33-0.74]; *P* < .001) in ALK-TKI-naïve patients.^8^ A recent retrospective analysis (multi-country) of the brigatinib early access program (EAP) population by Lin et al, showed that brigatinib was effective (7.5-10 months of median time to discontinuation [TTD]) and tolerable in real-world clinical practice, regardless of prior treatment with first- or next-generation (NG) ALK-TKIs.^17^ Another real-world retrospective analysis of the Latin American population in the EAP by Heredia et al showed similar positive trends for brigatinib.^18^

In general, there is a paucity of substantial real-world data for brigatinib. Outside of trial protocol mandated dosing, real-world dosing patterns of brigatinib have not been previously described. Given the evolving treatment landscape for ALK+ NSCLC, there is a pressing need for more data for brigatinib in both post-crizotinib and post-NG settings, which could help clinicians make informed decisions and maximize patient benefits. The objective of this study was to analyze the real-world treatment and dosing patterns of brigatinib in the US. We assessed characteristics, treatment patterns, adherence, discontinuation, and dosing patterns in patients with NSCLC receiving ALK inhibitors, with a focus on patients treated with brigatinib.

## Materials and Methods

This was a retrospective, non-interventional cohort study of patients who were prescribed brigatinib in clinical practice. The data were collected from IQVIA’s Longitudinal Patient-Centric Prescription Claims Database (LRx), which includes claims from 2001 for adjudicated prescriptions sourced from retail, mail-order, long-term care, and specialty pharmacies. It includes ~90% of all retail prescriptions and ~60% of all traditional and specialty mail-order prescriptions and covers over 1.4 billion prescriptions per year. It captures all prescriptions, including those fully paid by the consumer or insurance, and represents all payer types (cash, Medicare, Medicaid, and other third-party payers). LRx provides patient-level information on products/medications dispensed, quantity dispensed, days’ supply of the dispensing, age, gender, patients’ 3-digit zip code, specialty of the prescriber, Rx date filled, pharmacies, cost information, and payer type. All the data are HIPAA compliant to protect patient privacy.

The claims data from 1 April 2017 through 30 September 2020 were extracted from the LRx database. The index event date for each patient was defined as the date on which the first brigatinib claim was identified during the aforementioned selection window period. Based on the index event date, the pre- and post-index periods were defined for each patient: pre-index period spanned ≥12 months prior to the index event date, while the post-index period started after the index event date and continued until the end of the study or until the patient was lost to follow up.

Patients aged ≥18 years on the index event date, with at least one brigatinib claim during the index period, and with ≥12 months of pre-index and ≥1 month of post-index pharmacy stability (ie, the inclusion of data from pharmacies that consistently report data monthly) in the LRx database were deemed eligible for analysis. Patients identified with data quality issues (ie, missing age or sex) were excluded from the study.

Demographics, clinical characteristics, and the use of prior ALK-TKI treatments were assessed from the pre-index claims data. The following outcomes were collected during the post-index period: Medication Possession Ratio (MPR); adherence to index brigatinib therapy (MPR ≥80%); daily brigatinib pill burden; dose compliance score (DCS); discontinuation rate of index brigatinib therapy; TTD; probability of continued brigatinib therapy at 3, 6, and 12 months after initiation of therapy; and dosing patterns for brigatinib in terms of the percentage of patients who escalated to a dose of 180 mg/day or experienced dose reduction, time to dose reduction, and probability of continuing on brigatinib therapy at dose ≥180 mg/day and <180 mg/day (at maximum dose).

Analyses were conducted using SAS v9.4 (SAS Institute Inc., Cary, NC). Mean, median, and SD were generated as measures of central tendency and variance for continuous variables. For categorical variables, frequencies, and percentages were reported. Descriptive statistics were used to analyze baseline demographics and characteristics, and patterns for treatment, dosing, adherence, and discontinuations. The Kaplan-Meier (KM) curves were used to assess the TTD of brigatinib therapy and time to brigatinib dose reduction. The probability of continued therapy was evaluated using the survival probability (ie, probability of remaining on therapy) from KM analyses of TTD.

The study outcomes were defined as follows: (1) MPR—sum of the days’ supply for all claims of brigatinib during the length of therapy divided by the total number of days in the line of therapy, MPR was not capped at 100%. (2) Adherence—count and proportion of patients with MPR ≥80%. (3) Daily pill burden—total number of pills taken per day and calculated as the dispensed quantity/days’ supply. (4) DCS—sum of doses received from the first to the last prescription of ALK-TKI/ perfect compliance dose per day (per product label) × (days between first and last prescription + day supply of the last prescription). (5) Discontinuation of index brigatinib therapy—a gap of ≥90 days in brigatinib therapy or an initiation of a new ALK TKI. (6) TTD—number of months between initiation and discontinuation of brigatinib therapy. Patients who did not meet the definition of discontinuation were censored at the end of available follow-up. (7) Dose escalation—at least 1 claim for 180 mg pill strength or average daily dose of ≥180 mg/day. (8) Dose reduction—≥30 days of brigatinib supply with an average daily dose of either less than 180 mg/day (in patients who had reached ≥180 mg/day dose) or the maximum achieved dose (in patients who did not reach ≥180 mg/day dose). (9) Time to dose reduction—number of months from the start of target dose (ie, 180 mg/day) or maximum dose to the date of dose reduction. Patients who did not meet the criteria for discontinuation or switch and those in whom a dose reduction was not observed were censored at either end of the follow-up period or the end of the study period (whichever occurred first).

Subgroup analyses were performed by stratifying patients based on prior ALK-TKI therapy:

(1) Any prior ALK TKI: patients with at least one ALK TKI prior to brigatinib.
(2) Prior crizotinib and NG ALK TKI: patients with crizotinib followed by at least one NG ALK TKI (alectinib, ceritinib and lorlatinib) prior to brigatinib.
(3) Prior crizotinib and alectinib: patients with crizotinib followed by alectinib prior to brigatinib, and
(4) Prior alectinib only: patients with alectinib as the only ALK TKI prior to brigatinib.

In the dosing pattern analysis, patients were stratified based on the brigatinib dose: patients who reached ≥180 mg/day dose and patients who did not reach 180 mg/day dose.

## Results

**Figure 1.**
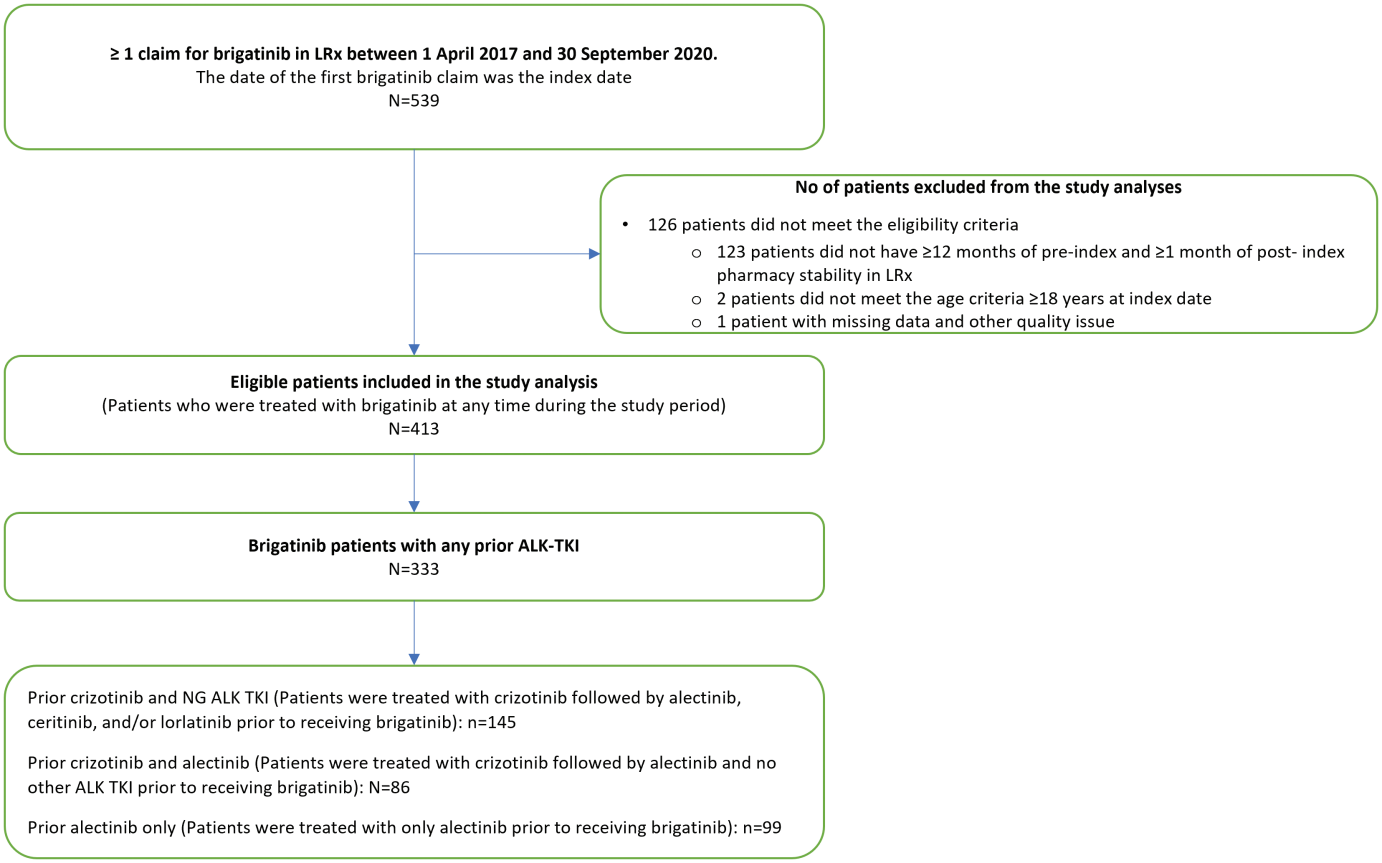
Patient attrition and study subgroups. Abbreviations: AKI-TKI, anaplastic lymphoma kinase-tyrosine kinase inhibitors; LRx, longitudinal patient-centric prescription claims; NG, next generation.

In total, records of 539 patients with ≥1 brigatinib claims were extracted from the LRx database, of which 413 met the eligibility criteria and were included in the analysis (Fig. 1). The mean (SD) age of eligible patients was 57.9 (12.9) years, and 58.4% were females. The third-party/commercial insurance was the most used payer (64.7%), followed by medicare part D (30.3%) (Table 1A).

**Table 1.** Baseline demographics and patient characteristics.

(A) Subgroups of patients stratified as per prior ALK-TKIs					
Characteristics	All brigatinib patients *N* = 413	Any prior ALK TKI^a^ *n* = 333	Prior crizotinib and NG ALK-TKI^b^ *n* = 145	Prior crizotinib and alectinib^c^ *n* = 86	Prior alectinib only *n* = 99
Age at index					
Mean (SD)	57.9 (12.9)	58.1 (13.0)	57.4 (13.0)	57.1 (12.9)	58.5 (11.9)
Median (interquartile range)	58 (18-85)	58 (18-85)	58 (21-85)	57 (21-85)	58 (21-85)
Gender, *n* (%)					
Male	172 (41.6%)	137 (41.1%)	57 (39.3%)	32 (37.2%)	46 (46.5%)
Female	241 (58.4%)	196 (58.9%)	88 (60.7%)	54 (62.8%)	53 (53.5%)
Region, *n* (%)					
Northeast	72 (17.4%)	60 (18.0%)	32 (22.1%)	23 (26.7%)	19 (19.2%)
Midwest	91 (22.0%)	74 (22.2%)	36 (24.8%)	21 (24.4%)	19 (19.2%)
South	99 (24.0%)	84 (25.2%)	33 (22.8%)	22 (25.6%)	30 (30.3%)
West	104 (25.2%)	83 (24.9%)	30 (20.7%)	12 (14.0%)	21 (21.2%)
Unknown	47 (11.4%)	32 (9.6%)	14 (9.7%)	8 (9.3%)	10 (10.1%)
Payer type, *n* (%)					
Third party	267 (64.6%)	220 (66.1%)	100 (69.0%)	64 (74.4%)	64 (64.6%)
Medicaid	13 (3.1%)	11 (3.3%)	1 (0.7%)	0 (0.0%)	7 (7.1%)
Medicare part D	125 (30.3%)	98 (29.4%)	44 (30.3%)	22 (25.6%)	25 (25.3%)
Cash	8 (1.9%)	4 (1.2%)	0 (0.0%)	0 (0.0%)	3 (3.0%)
Index year, *n* (%)					
2017	99 (24.0%)	82 (24.6%)	53 (36.6%)	26 (30.2%)	8 (8.1%)
2018	121 (29.3%)	103 (30.9%)	53 (36.6%)	40 (46.5%)	26 (26.3%)
2019	90 (21.8%)	68 (20.4%)	17 (11.7%)	9 (10.5%)	29 (29.3%)
2020	103 (24.9%)	80 (24.0%)	22 (15.2%)	11 (12.8%)	36 (36.4%)
Prior AKI-TKIs used, *n* (%)					
Crizotinib	212 (51.3%)	212 (63.7%)	145 (100.0%)	86 (100.0%)	0 (0.0%)
Ceritinib	67 (16.2%)	67 (20.1%)	51 (35.2%)	0 (0.0%)	0 (0.0%)
Alectinib	243 (58.8%)	243 (73.0%)	129 (89.0%)	86 (100.0%)	99 (100.0%)
Lorlatinib	20 (4.8%)	20 (6.0%)	12 (8.3%)	0 (0.0%)	0 (0.0%)
No prior ALK-TKI	80 (19.4%)	0 (0.0%)	0 (0.0%)	0 (0.0%)	0 (0.0%)
Follow-up time (months)					
Mean (SD)	11.9 (10.9)	12.3 (11.0)	14.3 (12.0)	14.2 (10.9)	8.0 (6.7)
Median (range)	8.4 (1.0-41.3)	8.5 (1.0-41.3)	10.3 (1.0-41.3)	12.3 (1.0-40.8)	6.8 (1.0-33.2)
(B) Subgroups of patients stratified as per brigatinib dose					
Characteristics	All brigatinib patients *n* = 413	Brigatinib patients who reach average daily dose ≥180 mg *n* = 318	Brigatinib patients who never reach average daily dose ≥180 mg *n* = 95	*P*-value^d^	
Age at index					
Mean (SD)	57.90 (12.94)	56.53 (12.63)	62.49 (12.98)	<.0001	
Median (interquartile range)	58 (18-85)	57 (18-85)	63 (28-85)	.0002	
Gender, *n* (%)					
Male	172 (41.6%)	142 (44.7%)	30 (31.6%)	.0233	
Female	241 (58.4%)	176 (55.3%)	65 (68.4%)		
Region, *n* (%)					
Northeast	72 (17.4%)	61 (19.2%)	11 (11.6%)	.081	
Midwest	91 (22.0%)	74 (23.3%)	17 (17.9%)		
South	99 (24.0%)	75 (23.6%)	24 (25.3%)		
West	104 (25.2%)	78 (24.5%)	26 (27.4%)		
Unknown	47 (11.4%)	30 (9.4%)	17 (17.9%)		
Payer type, *n* (%)					
Third Party	267 (64.6%)	212 (66.7%)	55 (57.9%)		
Medicaid	13 (3.1%)	12 (3.8%)	1 (1.1%)		
Medicare	0 (0%)	0 (0%)	0 (0%)		
Medicare part D	125 (30.3%)	90 (28.3%)	35 (36.8%)		
Cash	8 (1.9%)	4 (1.3%)	4 (4.2%)		
Unknown	0 (0%)	0 (0%)	(0%)		
Physician specialty, *n*(%)					
Oncology	359 (86.9%)	277 (87.1%)	82 (86.3%)	.8409	
General Practice	0 (0%)	0 (0%)	0 (0%)		
Other	54 (13.1%)	41 (12.9%)	13 (13.7%)		
Index year, *n* (%)					
2017	99 (24.0%)	80 (25.2%)	19 (20.0%)	.6809	
2018	121 (29.3%)	92 (28.9%)	29 (30.5%)		
2019	90 (21.8%)	70 (22.0%)	20 (21.1%)		
2020	103 (24.9%)	76 (23.9%)	27 (28.4%)		
Follow-up time (months)					
Mean (SD)	11.91 (10.91)	12.72 (10.91)	9.2 (10.52)	.0056	
Median (interquartile range)	8.37 (1.0-41.3)	8.67 (1.0-41.3)	6.37 (1.0-41.1)	.0001	

AKI-T ALK-TKI, anaplastic lymphoma kinase-tyrosine kinase inhibitors; NG, next generation.

Includes: alectinib, ceritinib, crizotinib, and lorlatinib.

Patients received crizotinib followed by any NG ALK-TKI prior to brigatinib.

Patients received crizotinib followed by alectinib and no other ALK-TKI prior to brigatinib.

*P*-value comparing patients treated with brigatinib who did and did not escalate to 180 mg/day dose.

Of the 413 patients treated with brigatinib, the majority received at least one prior ALK-TKI therapy (333 patients, 80.6%). Alectinib (58.8%) and crizotinib (51.3%) were the most used therapies prior to initiating brigatinib, while 16.2% of patients were treated with ceritinib and 4.8% used lorlatinib (Table 1A). A total of 145 (35.1%) patients received crizotinib followed by at least one NG ALK TKI prior to initiating brigatinib; of which 89.0%, 35.2%, and 8.3% also received alectinib, ceritinib, and/or lorlatinib, respectively (Table 1A). Eighty-six (20.8%) patients received crizotinib followed by alectinib prior to initiating brigatinib, and 99 (24.0%) had only alectinib (Fig. 1).

Overall, the median follow-up time from initiation of brigatinib was 8.4 months. The median follow-up period was longest in patients treated with prior crizotinib and alectinib (12.3 months) and shortest in patients who received prior alectinib only (6.8 months) (Table 1). Patients treated with alectinib only prior to brigatinib most commonly entered the study after 2018, whereas patients treated with prior crizotinib tended to enter the study prior to 2018 allowing for longer follow-up. Overall, the mean MPR score for brigatinib was 1.1, 92.7% of patients achieved adherence (MPR>0.8) and the mean DCS was 1.0. Similar trends were observed across all patients treated with brigatinib, regardless of the prior treatment with first or NG ALK-TKIs or the number and sequence of prior therapies (Table 2). Discontinuation of index brigatinib treatment was recorded in 167 patients overall (40.4%). The discontinuation rate was highest in patients who were pretreated with crizotinib and alectinib (51.2%), and lowest in the prior alectinib only group (32.3%). Among patients who discontinued brigatinib (*n* = 167), 100 patients (59.9%) had an additional ALK-TKI therapy: 57 patients (34.1%) switched to lorlatinib and 16 (9.6%) restarted brigatinib, while 13 (7.8%), 10 (6.0%), and 4 (2.4%) patients switched to alectinib, ceritinib and crizotinib, respectively.

**Table 2.** Adherence and dose compliance with brigatinib treatment

	All brigatinib patients *N* = 413	Any prior ALK TKI^a^ *n* = 333	Prior crizotinib and NG ALK-TKI^b^ *n* = 145	Prior crizotinib and alectinib^c^ *n* = 86	Prior alectinib only *n* = 99
MPR, mean (SD)	1.1 (0.4)	1.1 (0.4)	1.1 (0.3)	1.1 (0.4)	1.1 (0.5)
Adherent, %	92.7%	92.2%	91.0%	89.5%	90.9%
DCS, mean (SD)	1.0 (0.5)	1.0 (0.5)	0.9 (0.3)	0.9 (0.4)	1.1 (0.7)
Discontinuation rate, %	40.4%	41.7%	47.6%	51.2%	32.3%

ALK-TKI, anaplastic lymphoma kinase-tyrosine kinase inhibitors; DCS, dose compliance score; MPR, medication possession ratio; NG, next generation.

Subgroup of prior crizotinib cohort.

Subgroup of prior-crizotinib and NG ALK TKI cohort.

Patients received crizotinib followed by alectinib and no other ALK-TKI prior to brigatinib.

**Figure 2.**
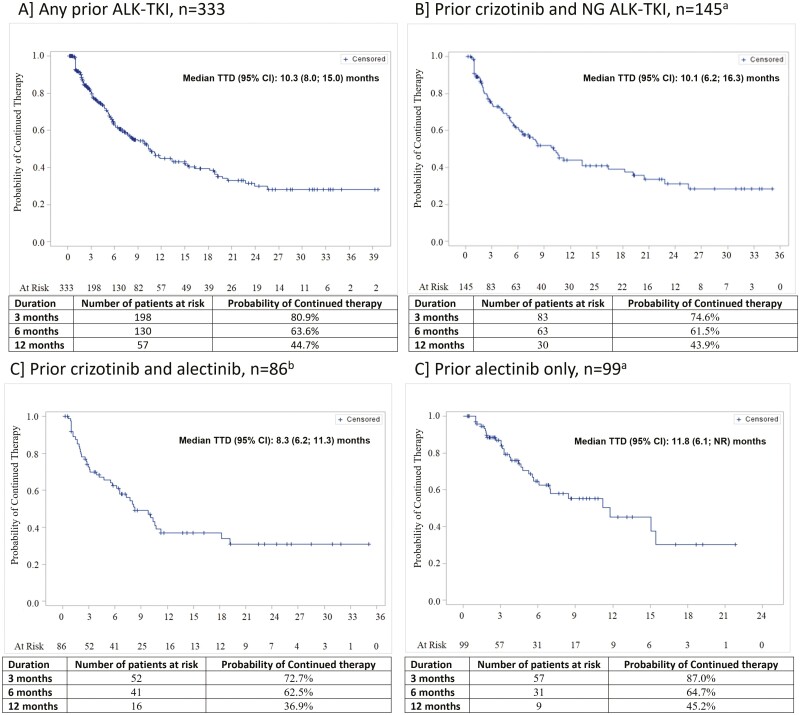
Time to brigatinib discontinuation. (A) Any prior ALK TKI, *n* = 333. (B) Prior crizotinib and NG ALK-TKI, *n* = 145. (C) Prior crizotinib and alectinib, *n* = 86. (D) Prior alectinib only, *n* = 99. Abbreviations: ALK-TKI, anaplastic lymphoma kinase-tyrosine kinase inhibitors; NG, next generation. aSubgroup of any prior ALK TKI cohort. bSubgroup of prior-crizotinib and NG ALK TKI cohort.

The median brigatinib TTD in the overall patient population (*N* = 413) was 10.3 months (95% CI, 8.2-15.0). The subgroup analysis showed that the median TTD was shortest in patients who received both crizotinib and alectinib (8.3 months [95% CI, 6.2-11.3]) and longest in the alectinib-only group (11.8 months [95% CI, 6.1-NA]) (Fig. 2A-D). Kaplan–Meier analysis suggested that 45% of patients treated with brigatinib remained on therapy at 12 months post-index. Patients who were pretreated with both crizotinib and alectinib demonstrated a lower probability of continued brigatinib therapy (36.9%) compared with other groups (43.9-45.2%) (Fig. 2A–D). The median TTD (95% CI) of the next-line ALK TKI post brigatinib was 7.2 (3.9-13.8) months. In patients who received lorlatinib after brigatinib was discontinued, the median (95% CI) lorlatinib TTD was 8.0 (3.9-NA) months.

**Figure 3.**
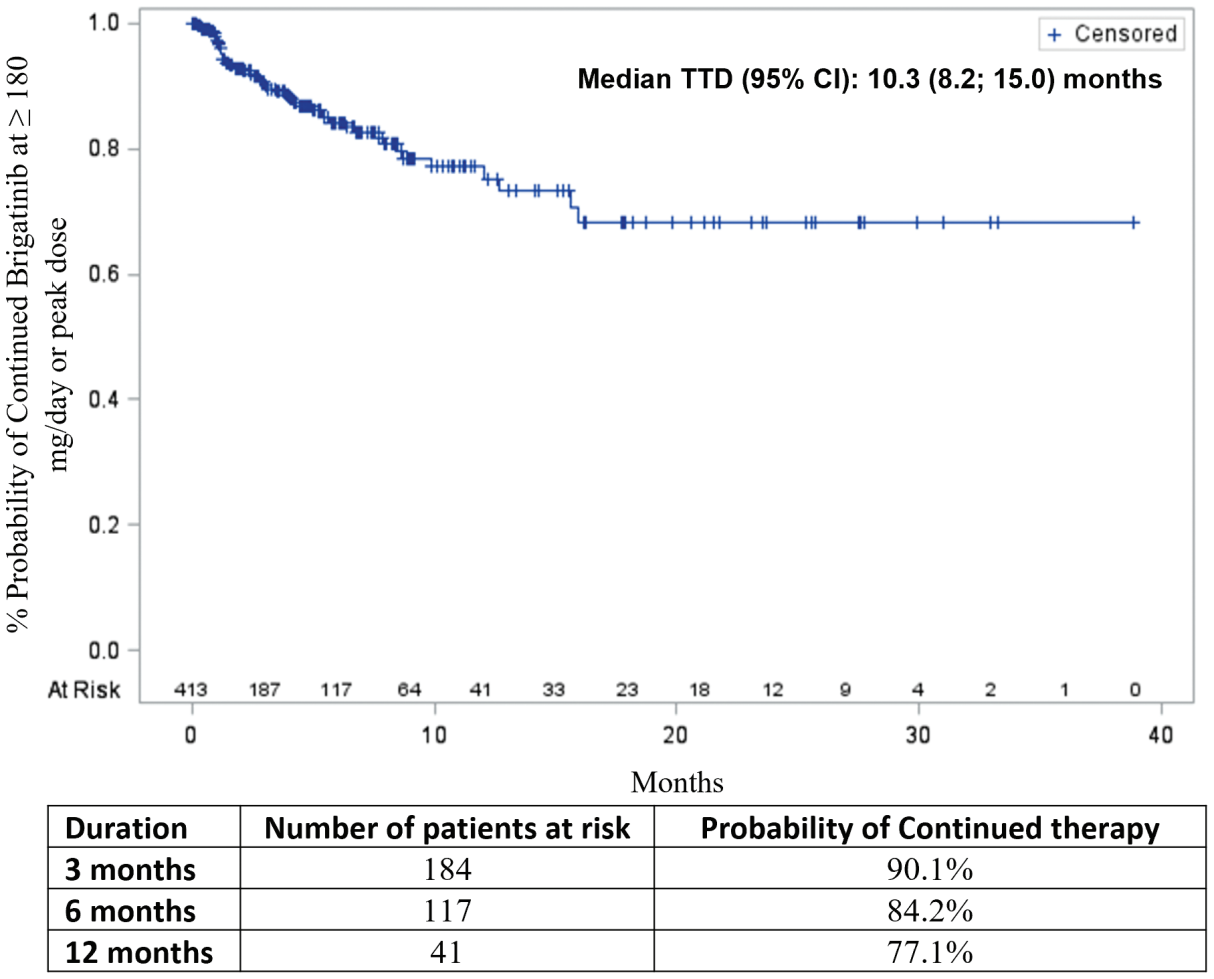
Time to brigatinib dose reduction (*n* = 413).

On average, patients took 2.7 pills per day. The daily pill burden decreased to 1.5 pills per day in patients who initiated treatment after the 180 mg pill became available in October 2017. This pattern was consistent across all subgroups based on prior ALK-TKI treatments. Most patients reached a brigatinib dose of 180 mg/day (318 [77.0%]). In all, 95 (23.0%) patients did not reach 180 mg/day. The subgroup of patients who did not reach 180 mg/day was relatively older, and had more females and a shorter follow-up period than the subgroup who achieved a dose of 180 mg/day (Table 1B). The most frequently observed maximum dose among patients who did not reach 180 mg/day was 90 mg (64.2%). Overall, dose reduction occurred in 49 (11.9%) patients, and the Kaplan-Meier analyses suggested that 90.1%, 84.2%, and 77.1% continued brigatinib at ≥180 mg/day or at peak at 3, 6, and 12 months, respectively. The median time to dose reduction was not reached (Fig. 3). Of the 318 patients receiving ≥180 mg/day dose, 42 (13.2%) had a dose reduction. Among these 42 patients, the majority (42.9%) tapered to a dose of 120 mg/day. The probability of continuing brigatinib at ≥180 mg/day was 89.3%, 84.6%, and 76.5% at 3, 6, and 12 months after dose escalation, respectively. Among the 95 patients who did not reach 180 mg/day, 7 had a dose reduction (to 120-30 mg/day brigatinib) from their peak dose. The probability of continuing brigatinib (at peak dose) was 93.8%, 81.1%, and 81.1% at 3, 6, and 12 months after the peak dose, respectively.

## Discussion

Using the LRx data, this study examined the real-world patterns in utilization and treatment sequencing among brigatinib-treated patients with ALK+ NSCLC in the US. As brigatinib was approved as a first-line therapy for NSCLC only recently (May 2020), the present study focuses on brigatinib use in the later-line treatment settings.^10^ Most of the patients in this study received ALK-TKIs (over 80%) prior to initiating brigatinib therapy. The most frequently used prior TKIs were alectinib and crizotinib, while the most frequent treatment sequence was crizotinib followed by at least one other ALK-TKI before initiating brigatinib. This trend was expected, as crizotinib was the first marketed ALK-TKI in the US (FDA approval in 2011), while alectinib was initially approved for post-crizotinib treatment in 2015 and received FDA approval for expanded use as a first-line agent in ALK+ NSCLC in 2017.^12^ Alectinib is currently the most frequently prescribed first-line ALK-TKI in patients with ALK+ NSCLC. The use of ceritinib is relatively low (16.2%), perhaps due to its high rate of gastrointestinal side effects.^19^ Treatment patterns for ALK-TKI usage and brigatinib sequencing in this study were aligned with the FDA approvals and access timings, as well as the approved indications, and were similar to a recent US-based real-world study,^20^ and studies on patients from the brigatinib early-access program (EAP).^17,18^

Overall, our study results demonstrated that, during the median follow-up period of 8.4 months, patients continued therapy for long durations (brigatinib TTD was 10.3 months). This trend was consistent across patient subgroups, regardless of prior ALK-TKI treatment and sequencing. TTD was comparatively shorter (8.3 months) in the heavily pretreated subgroup (crizotinib and alectinib). The short follow-up time (8.4 months) in our study and the recency of brigatinib’s US-FDA approval for ALK+ NSCLC (in 2017) may have influenced the TTD estimations. Also, the affordability of healthcare insurance would impact access to brigatinib treatment in the US population, and patients may discontinue treatment due to financial constraints, underestimating the TTD data. Overall, the TTD outcome in our study was consistent with a recent real-world study of brigatinib among patients previously treated with other ALK TKIs (TTD of 11 months was observed during the 27.5-month period of analysis).^17^ The probability of continued brigatinib therapy at 12 months in this study (43.5%) was consistent with a global real-world study (48.6%) and a study involving Latin American population (59.9%).^17,18^ After discontinuing brigatinib, most patients were treated with another ALK TKI and benefited from the subsequent treatments, suggesting that brigatinib could be used in earlier lines.

TTD was used as a surrogate endpoint for PFS to assess the effectiveness and treatment duration of brigatinib in our analyses, consistent with other real-world studies on ALK-TKIs.^17,18,20^ A correlation between TTD and PFS was observed in a post hoc analysis (*r = .*87) and a pooled analysis (*r = .*91) of clinical trials for metastatic NSCLC.^17,21^ Similarity in TTD and rwPFS (7.04 and 7.47 months, respectively) was also noted in a real-world evaluation, suggesting TTD’s importance as a pragmatic clinical endpoint.^20^ The randomized phase II ALTA study of brigatinib (180 mg/day dosing with a 7-day lead-in at 90 mg/day), showed a median PFS of >1 year (by investigator assessment, 15.6 months and by IRC assessment, 16.7 months) in patients with ALK+ NSCLC,^14,22-24^ which was longer than the TTD in our study. These variations in TTD and PFS outcomes between our real-world analysis and the ALTA clinical trial could be attributed to the fact that the ALTA trial had stringent eligibility criteria, excluding patients using any other ALK-TKIs (except crizotinib) or those receiving crizotinib within 3 days or chemotherapy or radiotherapy within 14 days of the first brigatinib dose, suggesting that the patients were not heavily pretreated. By contrast, over 80% of patients in the present study received at least 1 prior ALK TKI, including crizotinib, alectinib, ceritinib, and lorlatinib. Furthermore, our study population was more diverse, with variation in baseline characteristics and disease condition as expected among a real-world patient sample. It is likely that there are also differences in baseline sociodemographic, clinical, and prognostic factors of the patients. For example, our study only included patients from the US, whereas the ALTA trial was conducted in 20 countries. Furthermore, 66% of the ALTA study patients had a complete or a partial response at baseline perhaps leading to longer times on therapy and PFS compared to the present study as our real-world population likely included a lower proportion of patients with partial or complete treatment response at baseline (ie, patients in the present study may have been sicker at baseline and more likely to need additional lines of therapy sooner vs. ALTA patients). Furthermore, in the clinical scenario, measurement of response/progression depends on the physicians’ subjective judgment/interpretation, whereas in the randomized controlled trial, RECIST criteria, an objective, and well-defined method, is used to evaluate efficacy in oncology studies. Moreover, variability in imaging examination scheduling, treatment, and data collection methods could also influence the outcomes from the real-world analyses. The trend for shorter rwPFS (proxied by TTD outcome) in the present study versus PFS in prospective trials is in line with the published data for crizotinib. The rwPFS for crizotinib was reported to be 6.6 months, which was shorter than PFS of 7.7-11 months in the crizotinib trials. Differences between the real-world and clinical trial data have also been reported for ceritinib (rwPFS, 5.4 vs PFS, 5.4-6.9), and alectinib (rwPFS, 9.2 vs PFS, 8.3).^20^

Adherence and dose compliance for brigatinib were high in the overall population (93% of patients had an MPR>80%; median DSC of 1.0), with similar trends observed across patient subgroups. In line with this, most (84%) patients reached the recommended dose of ≥180 mg/day, suggesting that brigatinib was well-tolerated. However, this percentage is likely to be higher if patients were followed for a longer period. Kaplan-Meier analyses suggested that continuing therapy at ≥180 mg/day or at peak dose at 12-months was high (80-90%) in the present study. The TTD for brigatinib was longer in patients who did not reach 180 mg/day versus those who reached 180 mg/day (median duration: 23 months versus 10.6 months). The dose reduction rate for brigatinib was lower in our study (13.2%, at ≥180 mg/day dose) compared to the ALTA phase II study of crizotinib-refractory patients (29% of patients with a dose escalation) and the ALTA-1L study of ALK-TKI naïve patients (44% of patients).^8,14,22,25^ This variation in findings could be attributed to the differences between clinical trials and real-world settings. In the ALTA trials, the dose-reduction criteria were more stringent as they were investigator or protocol recommended as opposed to our less-regulated real-world study. Further, the asymptomatic laboratory abnormalities were the common causes of dose reductions in the ALTA trials. In ALTA-1L, the most common adverse events leading to dose reduction were increased blood creatine phosphokinase, increased lipase, and increased amylase (18%, 7%, and 4%).^25^ Outside of a clinical trial, these asymptomatic changes may not necessarily prompt dose reductions.

This study has several limitations and the results should be interpreted accordingly. The small sample size of patients treated with brigatinib limits the generalizability of the study observations. Though the LRx database covers a large proportion of all US claims, it does not include 100% of every patient’s claims nor does it include 100% of all specialty pharmacies where brigatinib and other ALK TKI therapies may be dispensed. As a result, the LRx data only include prescription data and may not include the patient’s full treatment journey, including the prior treatments (eg, non-oral chemotherapy and non-oral immunotherapy). Not all treatments including chemotherapy and radiotherapy were captured in the database. Also, the patients may obtain ALK-TKIs outside of the pharmacies included in LRx database, in which case, the related treatment data would be missing. Therefore, it is possible that some patients may be misclassified as “discontinued” due to missing data underestimating the TTD results. Further, the information required for dosing analyses may not be accurately captured in the LRx data. Also, the claims (billing and reimbursement) data do not provide information on reasons for discontinuation/dose modifications/non-escalation. Despite these limitations, we believe, that our study adds to the understanding of real-world brigatinib use. The data were generalizable to the overall US population and are payer agnostic. Unlike analyses conducted in adjudicated claims databases, there was no under-representation of patients >65 years of age.

## Conclusion

This US-based real-world study provides an early insight into the effectiveness and tolerability of brigatinib with low dose reduction rates and high adherence to therapy. Brigatinib appears to be effective and well-tolerated in the real-world ALK+ NSCLC population in the US, showing benefit in patients after a next generation ALK-TKI. Notably, dose reduction rates appeared markedly less than those seen in trials when most trial-related dose reductions were for asymptomatic laboratory abnormalities.

## Data Availability

The data that support the findings of this study are available from IQVIA but restrictions apply to the availability of these data, which were used under license for the current study, and so are not publicly available.
